# Randomized clinical trial to compare efficacy and safety of repeated courses of rituximab to single-course rituximab followed by maintenance mycophenolate-mofetil in children with steroid dependent nephrotic syndrome

**DOI:** 10.1186/s12882-020-02153-5

**Published:** 2020-11-30

**Authors:** Biswanath Basu, Stella Preussler, Anja Sander, T. K. S. Mahapatra, Franz Schaefer

**Affiliations:** 1 Division of Pediatric Nephrology, Department of Pediatrics, Nilratan Sircar Medical College & Hospital, Kolkata, West Bengal 700014 India; 2 Institute of Medical Biometry and Informatics, University of Heidelberg, Im Neuenheimer Feld 130.3, 69120 Heidelberg, Germany; 3 Department of Pediatrics, Nilratan Sircar Medical College & Hospital, Kolkata, West Bengal 700014 India; 4 Division of Pediatric Nephrology, Center for Pediatrics and Adolescent Medicine, University of Heidelberg, Im Neuenheimer Feld 430, 69120 Heidelberg, Germany

**Keywords:** Rituximab, Mycophenolate-Mofetil, Childhood nephrotic syndrome, Steroid dependent nephrotic syndrome

## Abstract

**Background:**

Approximately 30% of children with idiopathic nephrotic syndrome develop a complicated course with frequent relapses or steroid dependency. Rituximab, a B cell depleting monoclonal antibody, is a safe and effective alternative to steroids or other immunosuppressants for achieving and maintaining remission in this population at short term. Despite the good initial response relapses inevitably occur after regeneration of B lymphocytes, necessitating either repeat courses of rituximab or addition of another steroid-sparing immunosuppressant.

**Methods:**

This is a prospective, single-center, open-label, two-parallel-arm randomized controlled phase III study among children with steroid dependent nephrotic syndrome who are maintained in remission with oral steroids. One hundred children will be randomized to either Rituximab and maintenance Mycophenolate mofetil (A) or repeated courses of prophylactic Rituximab only (B). In arm A, mycophenolate mofetil (1200 mg/m^2^ per day) will be started 3 months after Rituximab administration. In arm B, Rituximab infusions will be administered at 0, 8 and 16 months if B cell count normalize at the given time points. Prednisolone will be discontinued in both groups 2 weeks following first course of rituximab. Primary aim is to evaluate the difference in 24-month relapse-free survival. Main secondary endpoints are cumulative prednisolone dose, frequency of relapses and changes in anthropometry. Circulating B lymphocyte populations will be studied as biomarkers or predictors of rituximab responsiveness and adverse events will be analysed.

**Discussion:**

The study will provide evidence as to the comparative safety and efficacy of two alternative steroid-sparing therapeutic options in children suffering from steroid dependent nephrotic syndrome. The two-year study design will address the long-term results obtained with the alternative treatment protocols.

**Trial registration:**

This trial was prospectively registered to the Clinicaltrial.gov (NCT03899103 dated 02/04/2019; https://clinicaltrials.gov/) and Clinical Trials Registry of India (CTRI/2019/04/018517 dated 09/04/2019).

## Background

Whilst idiopathic nephrotic syndrome in children usually responds well to corticosteroid treatment, more than two thirds of patients experience relapses and 30% develop a complicated course with frequent relapses or steroid dependency (SDNS) [1–7]. Relapses may lead to serious complications from anasarca, infections, thrombosis and malnutrition. Repeated or even continuous steroid treatment leads to considerable medication related toxicity and morbidity [1–6].

Hence, the primary aim of treatment is to reduce the number of relapses, the cumulative dose of corticosteroids, and the incidence of serious complications. Several prospective studies have suggested that Rituximab, a B-lymphocyte depleting monoclonal antibody, could be a safe and effective alternative to steroids and conventional steroid-sparing immunosuppressants such as calcineurin inhibitors or mycophenolate-mofetil (MMF) to achieve and maintain remission in this population [7–11]. Single rituximab infusions reliably suppress disease relapses for 6 to 12 months with a very mild side effect profile [7–11]. In the RITURNS trial we demonstrated a significant and clinically relevant reduction of relapse rates by primary use of Rituximab as compared to standard CNI therapy during a 12-month observation period, accompanied by a more favourable side effect profile [7]. However, relapses inevitably occur during extended follow-up following regeneration of B-lymphocytes. Therefore, further modification of Rituximab treatment, including repeated courses of Rituximab or adjunct immunosuppressive therapies, may be necessary for maintaining long-term remission. A few case series suggested that maintenance therapy with MMF after rituximab administration was effective for maintaining long-term remission in children with complicated nephrotic syndrome, with a largely benign side effect profile [12–14]. The long term follow up of our RITURNS trial also revealed improved relapse free survival with maintenance MMF therapy following rituximab re-exposure [14]. Hence, repeated courses of Rituximab or sequential maintenance therapy with MMF following the initial Rituximab course have been proposed as long-term treatment options in SDNS [7–11]. Our study will compare the relative efficacy and safety of these protocols over a 2-year period.

## Methods/design

### Aim, design and setting of the study

The RITURNS II trial is a prospective, single-center, open-label, two-parallel-arm randomized controlled phase III study. The aim is to evaluate the efficacy and safety of repeat courses of Rituximab to that of maintenance MMF following a single course of Rituximab in maintaining remission over 24 months in children with SDNS (as defined in Table 1). The test hypothesis is as follows: prophylactic repeated courses of Rituximab will result in improved relapse free survival compared to maintenance Mycophenolate Mofetil following single course of Rituximab infusion in children with steroid dependent nephrotic syndrome (SDNS), i.e., testing superiority.

**Table 1:** Standard definitions for nephrotic syndrome used in this document

*Remission*	Urine albumin nil or trace (or proteinuria < 4 mg/m^2^/h or uPCR < 200 mg/g (< 20 mg/mmol)) for 3 consecutive early morning specimens
*Relapse*	Urine albumin 3+ or 4+ (or proteinuria > 40 mg/m^2^/h or uPCR ≥2000 mg/g (≥200 mg/mmol)) for 3 consecutive early morning specimens, having been in remission previously
*Steroid dependence*	Two consecutive relapses when on alternate day steroids or within 14 days of its discontinuation

*uPCR* Urine protein creatinine ratio

The trial was started on 15th May, 2019 at the Division of Pediatric Nephrology, Department of Pediatrics, NRS Medical College & Hospital, Kolkata, India. By 15th September 2020, 70 patients have been enrolled in the trial.

### Characteristics of participants

Inclusion criteria: children between 3 and 16 years with SDNS; minimal change disease/FSGS/MesPGN as per kidney biopsy report; estimated glomerular filtration rate (eGFR) > 80 ml/min per 1.73 m^2^ at study entry; remission at study entry (urine albumin nil or trace (or proteinuria < 4 mg/m^2^/h or uPCR < 200 mg/g (< 20 mg/mmol)) for 3 consecutive early morning specimens); not received any steroid sparing agent (including levamisole, calcineurin inhibitors, mycophenolate mofetil, cyclophosphamide, mizoribine, chlorambucil or rituximab) previously; parents willing to give informed written and audiovisual consent; and ability to swallow tablet.

Exclusion criteria: known etiology (e.g., lupus erythematosus, IgA nephropathy, amyloidosis, malignancy, other secondary forms of NS); patients with severe leukopenia (leukocytes < 3.0× 1000 cells/mm^3^), severe anemia (haemoglobin < 8.9 g/dl), thrombocytopenia (platelet < 100.0 × 1000 cells/mm^3^) or deranged liver function tests (AST or ALT to > 50 IU/L) at enrolment; known active chronic infection (tuberculosis, HIV, hepatitis B or C); and live vaccination within 1 month prior to screening.

### Processes, interventions and comparisons

#### Participant screening and recruitment

Consecutive cases of SDNS presenting at the study centre will be screened for eligibility. A preliminary interview for clinical and pharmacological history will be performed in order to verify the eligibility criteria. All participants undergo kidney biopsies before enrolment in the trial. A study investigator will explain the project, deliver information sheet. Children fulfilling the inclusion criteria will be recruited. The Pediatric Nephrology Division of NRS Medical College & Hospital, Kolkata is one of the largest dedicated pediatric nephrology services at Government Sector in India. An average of 140–180 children are seen per week in the nephrotic syndrome outpatient clinic; approx. 20–30% of these are new cases and about 60–80% of new cases are SDNS.

#### Interventions

##### Arm a (rituximab and mycophenolate mofetil, control treatment)

First Course Rituximab at Randomization: Two infusions will be administered intravenously at a 7-day interval at standard dose (rituximab 375 mg/m^2^, maximum 500 mg). Circulating B cells will be measured 24 h after second rituximab administration. If B cell count exceeds 5/mm^3^, it will be measured again after 1 week. If count is still > 5/mm^3^, one more dose of rituximab will be administered.

Co-intervention with Rituximab at Randomization: Prednisolone will be continued at alternate-day doses for 2 weeks (1.5 mg/kg (max.40 mg) per 48 h in patients on daily prednisolone at time of randomization; pre-randomization dose in those already on alternate-day dosing). At 2 weeks, prednisolone will be discontinued.

Addition of Maintenance MMF from month 4 onwards: Maintenance oral MMF 1200 mg/mt^2^ daily in two divided doses will be added from month 4 of follow-up and continued over the remaining study period unless an adverse event occurs.

##### Arm B (repeated courses of rituximab only, experimental treatment)

First Course Rituximab at Randomization: Same as Arm A.

Co-intervention with Rituximab at Randomization: Same as Arm A.

Prophylactic 2nd and 3rd Course Rituximab Re-administration at 8 Months and 16 Months of Follow-up: Prophylactic 2nd and 3rd course rituximab re-administration will be done at 8 months and 16 months of follow-up if B cell count normalize (please refer to Additional file 1: Appendix 0 for our reference range of normal age specific B cell count) and patient is in remission. Dose and route will remain as explained in Table 2.

**Table 2:** Intervention in both arms over time

	Group A (RITUX +MMF)	Group B (Repeated RITUX only)
At randomization	First course rituximab. Stop steroid at 14 days.	First course rituximab. Stop steroid at 14 days.
At month 4	Add MMF and continue over study period unless any adverse event	–
At month 8	–	2nd course prophylactic rituximab if B cell normalize.
At month 16	–	3rd course prophylactic rituximab if B cell normalize.
At month 24	FOLLOW-UP COMPLETED	
	Any relapse during the observation period will be treated with prednisone (2 mg/kg/day) until remission, followed by 1.5 mg/kg alternate day for 1 month.	
If > = 2 relapses during any 6 month period, treat by	2nd course rituximab if B cell in normal range. Continue MMF.	Add MMF.

#### Comparisons, randomization & blinding

Treatment arm A will be compared to treatment arm B. Randomization will be performed 1:1 using stratified block randomization with varying block sizes and including age (≤ 7 vs. > 7 years) and renal histology (MCD vs. FSGS) as stratification factors. To achieve comparable intervention groups and to minimize a potential selection bias, patients will be allocated in a concealed fashion by means of randomization after enrolment. To that end, a computer generated random list will be created according to the stratification factors and block sizes and sealed opaque envelopes will be provided by an independent data manager from the Institute of Medical Biometry and Informatics, University of Heidelberg. Enrolment and assignment of the participants to interventions will be done by investigators and resident doctors at NRS Medical College & Hospital, Kolkata, India. The trial will be open-label with no masking of patients or study staff to the treatment allocation.

#### Study visits & assessment

##### Baseline assessment at enrolment

Retrospective clinical information will be obtained from the case records and clinic files. This will include information regarding age of onset of disease, disease type, duration of total disease etc.; and treatment received, number of relapses, cumulative steroid dose, detailed anthropometry and investigations during the last 12 months prior screening. The patients shall be clinically screened for significant infection. The information will be entered into the patient data sheet (refer to Additional file 1: Appendix 1). Clinical examination shall be done and data shall be recorded.

##### Follow-up visit and drug compliance

Study visits will be scheduled at enrolment, then weekly for the first month, then at 4th month and then 4 monthly until the end of the study or during relapse, remission and if there is any specific need after enrolment. In Arm B (repeated courses of rituximab only), there will be 2 extra visits 1 week after the prophylactic 2nd and 3rd courses of rituximab re-administration at 8 months and 16 months, respectively.

Complete blood count, kidney function, liver enzymes, serum electrolytes, plasma proteins, serum cholesterol, serum albumin, serum immunoglobulin and B lymphocyte count as applicable will be obtained during protocol visits and in between period if needed. Circulating B-cell count (number/mm^3^) will be measured at enrolment, then every fortnight for the first month, then at 4 months and then 4-monthly until the end of the study or during relapse and if there is any specific need after enrolment. An overview is given in Table 3. At the 12- and 24-month visits a quality of life (QoL) assessment together with a specific questionnaire concerning the impact of the different treatment patterns on patient and family life shall be performed.

**Table 3:** Participants time line and follow up data sheet over study period

	−7 days	0 days	7 days	14 days	21 days	1 month	4 months	8 months	8 months 1 wk	12 months	16 months	16 months 1 wk	24 months	Close out
BMI for age Z score	Enrolment	Allocation & intervention	–	–	–	–			–					
Height for age Z score			–	–	–	–			–					
If on Oral Steroid, Dose (Mg/kg alternate day)														
Cumulative prednisolone dose during study period (mg/kg)			–	–	–	–	–	–	–					
If on MMF, dose (mg/kg)														
Number of Rituximab injection received														
Total B Lymphocyte count:														
Any Relapse during this period														
Time to first relapse (weeks)														
Albumin (g/dl)			–		–									
Urine protein-creatinine ratio			–		–									
Blood leucoocyte count (n/mm^3^)			–		–	–								
Blood leucocyte differential count (n/mm^3^)			–		–	–								
Blood Platelet count (n/mm^3^)			–		–	–								
Haemoglobin (g/dl)			–		–	–								
Serum Sodium (meq/l)			–		–	–	–		–					
Serum Potasium (meq/l)			–		–	–	–		–					
Serum Calcium (mg/dl)			–		–	–	–		–					
Aspartate transaminase (IU)			–		–	–	–		–					
Alanine transaminase (IU)			–		–	–	–		–					
Alkaline phosphatase (IU)			–		–	–	–		–					
Fasting blood glucose (mg/dl)			–		–	–	–		–					
Immunoglobulin exam														
Cholesterol (mg/dl)			–		–									
Estimated GFR, ml/min/1.73 m^2^			–		–									
Use of any other drugs														
Clinical exam (if specific abnormal –describe below) (Hypertension-Y/N)														
Any adverse report yes/no (if yes –describe in full detail)														
Study discontinuation or withdrawal (If Yes weeks of discontinuation & reason for discontinuation) Treatment related (yes/no)														

REMARKS:

ADVERSE EVENT

At each visit, the drug will be handed over to the parent/guardian in an amount sufficient to last the interim duration; ten extra doses would be provided to ensure compliance even if follow-up is delayed for some reason. At the follow-up visit, empty packs of drug provided in previous visit would be collected and pill count done to check compliance to intervention. At discharge from the Nephrology Unit, each patient will receive a clinical diary, to be filled with dipstick proteinuria levels and current treatment. Adherence will also be recorded in the patients’ diary.

##### Duration of study

**Fig. 1:**
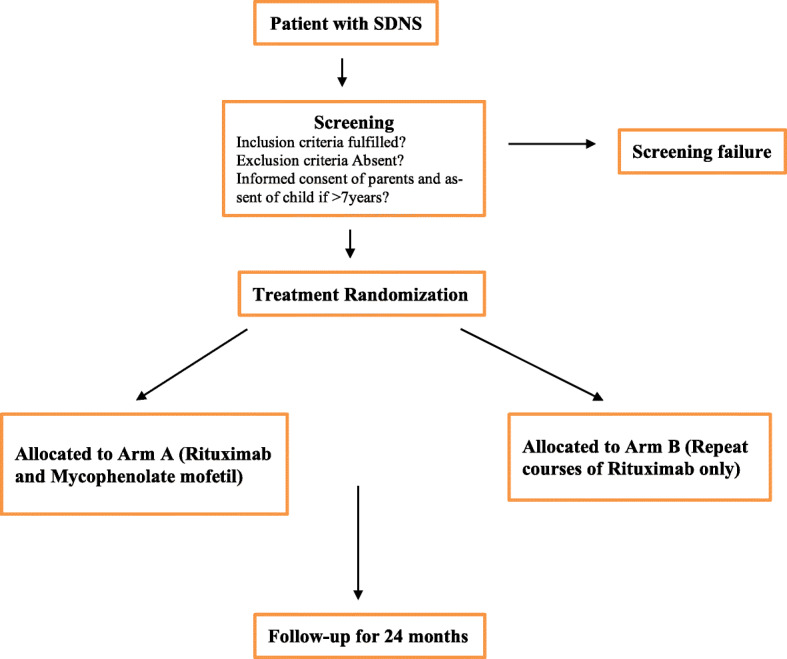
Study flow chart

The overall duration of the trial is expected to be approximately 36 months. Recruitment of the patients is planned over a time period of 12 months, and the duration of follow-up per patient (start at treatment initiation) will be 24 months (Fig. 1).

#### Standard case management

##### Relapse management

Relapses will be treated by reinstitution of daily therapy with prednisolone (2 mg/kg/day, maximum 60 mg) until remission, followed by alternate-day dosing (1.5 mg/kg, maximum 40 mg) for 4 weeks, and then stopped.

##### Other co-interventions

Rituximab will be administered after proper premedications (administer 30 min prior to rituximab) with oral paracetamol, oral diphenhydramine and intravenous hydrocortisone as per center practice.

Hypertension, defined as blood pressure > 95th percentile (for age, height and sex) in those previously normotensive, shall be treated at the discretion of the investigator. Children shall be treated with oral calcium+vitamin D when on oral prednisolone. Other medications shall be used at the discretion of the treating physician. Trimethoprim-sulfamethoxazole shall be administered from the beginning of rituximab treatment until recovery of peripheral blood B cell for prophylaxis against *Pneumocystis jirovecii* infection. The investigator will record all concomitant medications taken by the subject during the study from the date of informed consent, in the appropriate section of the case report form.

#### Safety data

We will collect any untoward medical occurrence in the form of signs, symptoms, abnormal laboratory findings, or diseases that emerges or worsens relative to baseline (i.e. present at the initial study visit). All adverse events including any transfusion reaction following rituximab infusion will be graded according to the Common Terminology Criteria for Adverse Events, version 3 [15]. Policy and approach to define adverse events is reported in Additional file 1: Appendix 2.

#### Study termination

Subjects will be informed that they have the right to withdraw from the study at any time, without prejudice to their medical care, and that they are not obliged to state their reasons. The time and reason of treatment discontinuation will be documented in the CRF. Independent data-safety and monitoring board (DSMB) (or IRB) will monitor the study progress at regular interval.

#### Data management & quality assurance

The investigators will record study data in the case report form (CRF). The clinical and laboratory data shall be entered in electronic format. At monthly intervals, the data entries will be checked for completeness and the files reviewed for errors.

At the end of the study, the data will be transformed into different data formats for archiving and to ensure that it can be reused. It is planned to make the trial data on which scientific publications are based, as well as all the primary data, publicly available in an appropriate online data repository to allow re- and meta-analyses after completion of the trial.

### Study endpoints, estimand definition, power calculation and type of statistical analysis used

#### Study endpoints

##### Primary endpoint

The primary endpoint is the time from treatment initiation to first relapse or death (whichever occurs first).

##### Secondary endpoints

Main secondary endpoint is 1.) the cumulative prednisolone requirement (mg/kg/yr) over the first 12 and 24 months, respectively. Further secondary endpoints are 2.) at least one relapse within the first 12 months (yes/no); 3.) at least one relapse within 24 months (yes/no); 4.) occurrence of two or more than two relapses in any period of 6 months within 24 months (yes/no); 5.) occurrence of four or more relapses in a period of any 12 months within 24 months (yes/no); 6.) at least One episode of life threatening infection or severe relapse (anasarca with hypovolemia or thrombosis) requiring hospital admission within any 12 months (within 24 months) and 24 months (yes/no), respectively; 7.) impairment of renal function (eGFR < 30 ml/min per 1.73 m^2^ or loss of eGFR by > = 30% with regard to baseline) within any 12 months (within 24 months) and 24 months (yes/no), respectively; 8.) t*reatment failure* (yes/no): defined as composite endpoint consisting of the three endpoints as defined above under 5., 6. and 7. referring to month 24; 9.) number and severity of adverse events; 10.) eGFR at 4, 8, 12, 18 and 24 months, respectively; absolute change in eGFR from baseline to 4, 8, 12, 16 and 24 months, respectively; 11.) number of relapses within months 0–24, 0–12 and 12–24, respectively; 12.) number of different steroid toxicity events (new onset) within months 0–24; 13.) off steroids at month 24 (yes/no); 14.) abnormal values in biochemical tests and haematology assessments (yes/no) according to the Common Terminology Criteria for Adverse Events, version 3 [15]; 15.) height SDS at 12 and 24 months, respectively; 16.) absolute change in height SDS from baseline to 24 months; 17.) BMI SDS at 12 and 24 months, respectively; 18.) absolute change in BMI SDS from baseline to 24 months; 19.) total B Lymphocyte count (% and per mm^3^) over time.

#### Estimands

An estimand is defined through the treatment, the targeted population, the variable, a specification of how to handle intercurrent events (postrandomization events) and a population-level summary [16]. In the following, the primary estimand corresponding to the primary objective, as well as the main secondary estimands corresponding to the main secondary objective are described.

##### Primary Estimand

The treatment is described as above and the targeted population is defined through the in- and exclusion criteria who received at least one dose of study treatment. The intercurrent events are handled as follows. Administrative censoring at the end of study, withdrawing and loss to follow-up without prior deterioration are assumed to be uninformative and will be censored. This implies the assumption that censored patients would have behaved as the uncensored patients. Hence, this describes a scenario in which the intercurrent event would not occur (hypothetical strategy). It is assumed that the event death is worse than a first relapse, therefore it is included in the definition of the variable (composite strategy). Deviations from the treatment protocol and treatment switching will be ignored (treatment policy strategy). The population-level summary measure is the hazard ratio.

##### Main secondary Estimands

Treatment and population are specified as for the primary estimand. The variable is defined as the amount of cumulative prednisolone requirement (mg/kg/year) over 12 and 24 months, respectively. Intercurrent events are handled as follows. Withdrawals, lost to follow-up, death, treatment switching, deviations from the treatment protocol and AEs will be ignored (treatment policy strategy). The summary measure is the difference in variable means.

#### Sample size/ power calculation

The sample size calculation is based on the primary endpoint, time to first relapse or death (whichever occurs first), using log-rank test to compare the event times of arm A and B. An event rate of 25% after 24 months in arm A and 5% in arm B was assumed (hazard ratio = 0.178) [12–14, 17–22]. Assuming an individual follow-up time of 24 months, in total 95 patients (15 events) are required to prove efficacy with a power of 90% and a two-sided alpha level of 5% for the log-rank test. To account for major protocol violations and drop-outs an assumed drop-out rate of 10% yields a total of 100 patients to be randomized [23, 24].

#### Statistical analysis

The analysis will be performed on the full analysis set (FAS; according to the intention to treat principle), the per-protocol set and the safety analysis set (for details refer to Additional file 1: Appendix 3).

All documented variables will be analyzed descriptively by tabulation of empirical distribution measures accordingly to the scale level and under specification of the number of non-missing values.

The confirmatory primary analysis will be based on the FAS. The primary endpoint will be compared between the two treatment groups using a two-tailed logrank test stratified by age (≤ 7 vs. > 7 years) and renal histology (MCD vs. FSGS) at an overall type I error rate of 0.05. The survival curves will be estimated using the Kaplan-Meier product-limit method, and the corresponding confidence intervals will be calculated using Greenwood’s formula [25]. Missing values on the covariates will be imputed by a multivariate imputation model. To assess the impact of major protocol deviations, an analogous analysis of the primary endpoint will be performed on the per protocol set. In addition, the possible influence of age (continuous) and renal histology on the primary endpoint is evaluated within a multivariate Cox regression analysis including treatment group (under the assumption of proportional hazards) performed on the FAS. Furthermore, a multivariate Cox regression analysis with covariates, age (continuous), sex, renal histology (MCD vs. FSGS), duration of the nephrotic syndrome (years), number of relapses in previous year and group will be performed on the FAS. All secondary endpoints will be analyzed based on the FAS and the results will be interpreted descriptively.

The **s**afety analysis will be based on the safety analysis set and includes calculation and comparison of frequencies and rates of adverse and serious adverse events reported in the two treatment groups. Furthermore, the relation of AEs with the cumulative dose of Rituximab, MMF and Prednisolone will be described, respectively.

More details can be found in the statistical analysis plan which will be finalized prior to database closure and before performing any analyses. All analyses will employ SAS Version 9.4 or higher.

## Discussion

Existing clinical practice guidelines recommend calcineurin inhibitors (CNI) as first-line steroid sparing therapy for children with SDNS, whereas Rituximab is used as a rescue for CNI resistant cases [7–11]. However, the excellent efficacy and safety profile of Rituximab raised the question whether it could be used as a first-line alternative to CNI therapy. In the RITURNS trial we demonstrated a significant and clinically relevant reduction of relapse rates by primary use of Rituximab as compared to standard CNI therapy during a 12-month observation period, accompanied by a more favourable side effect profile [7]. To further assess the long-term efficacy and safety of the treatment protocols we extended the follow-up of the RITURNS trial cohorts by another 2 years [14]. In this follow-up study we noted disease recurrence in all patients of the Rituximab arm, with first relapses occurring between 6 and 24 months following Rituximab exposure. This finding supports existing literature on rituximab in children with nephrotic syndrome [26–28].

The transient nature of the therapeutic effect of Rituximab creates a need to develop safe and efficient longer-term treatment protocols for SDNS. Repeated prophylactic Rituximab administration is one therapeutic option. Alternatively, an oral maintenance steroid-sparing immunosuppressant can be initiated before B cells are repleted. Encouraging results have been reported for both approaches in retrospective case collections and small cohort studies [12–14, 17–22].

Rituximab re-treatment without any maintenance immunosuppression prolonged the relapse-free survival in previously relapsing patients [19–22]. In the RITURNS trial we demonstrated that B cell counts recovered by 12, 39 and 93% at 6 mo, 9 mo and 12 mo respectively following a single course of rituximab and all relapses occurred beyond 8 months after administration in patients who had achieved full B-cell recovery [7]. Two other trials demonstrated a similar pattern of B-cell depletion following rituximab [26–28]. We chose an 8-monthly re-treatment schedule as a compromise to avoid unnecessary and costly over-immunosuppression on the one hand and extended periods of fully recovered B cell activity at risk of relapses on the other hand.

MMF maintenance treatment following Rituximab re-exposure in post-Rituximab relapsers also extended the duration of remission, with two thirds of patients remaining relapse-free for 2 years [12]. The long term follow up of our RITURNS trial also revealed better relapse free survival with maintenance MMF therapy following rituximab induction, with a relatively benign side effect profile [14]. A recent retrospective multicenter study revealed that children receiving low-dose rituximab without maintenance immunosuppression had the shortest relapse-free survival, and both the cumulative rituximab dose and maintenance immunosuppression had important effects on treatment outcomes [29].

These observations and considerations motivated us to design a trial comparing these alternative treatment approaches.

RITURNS II will be the first randomised clinical trial comparing the effects of extended B cell depletion by repeated rituximab administration to that of maintenance MMF after a single course of Rituximab in maintaining extended disease remission in children with SDNS. The sample size and evaluation period will be sufficient to empower the assessment not only of differences in therapeutic efficacy but also to obtain valid safety information. The lack of the long term safety data is the main current concern of clinicians precluding extensive use of Rituximab in children with SDNS.

Another strength of this trial will be the exploration of potential markers and mediators of treatment efficacy and adverse effects. We will regularly monitor B-lymphocyte counts, as well as oral drug adherence using patient diaries and returned pill counting.

Potential limitations of our study are given by its single-centre nature, and the lack of blinding which would have required additional administration of placebo tablets and infusions, a procedure that was not deemed acceptable in this vulnerable population of children, nor required given the fact that urine protein measurement is an objective endpoint that is hardly affected by investigator expectation bias. Bias from potential confounding factors will be minimized addressed by inclusion as covariates into the statistical analysis.

In conclusion, we perform this randomized clinical trial to compare efficacy and safety of repeated courses of rituximab to that of maintenance mycophenolate mofetil following a single course of rituximab in maintaining remission over 24 months among children with SDNS, to provide an evidence base for the long-term management of children suffering from complicated nephrotic syndrome.

## Supplementary Information

**Additional file 1.**

## Data Availability

The datasets generated and/or analysed during the current study will be made available in an appropriate online data repository. All investigators will have access to the final trial dataset.

## Abbreviations

AE: Adverse Event
BMI: Body mass index
BP: Blood pressure
BSA: Body surface area
CRF: Case Report Form
DSMB: Data safety and monitoring board
EC: Ethics Committee
eGFR: estimated glomerular filtration rate
FAS: Full Analysis Set
FRNS: Frequent-relapse nephrotic syndrome
FSGS: Focal segmental glomerulosclerosis
GCP: Good Clinical Practice
ITT: Intention-to-Treat
MCD: Minimal Change disease
MesPGN: Mesangioproliferative glomerulonephritis
MMF: Mycophenolate mofetil
PI: Principal Investigator
PP: Per-Protocol
Q: Quarter (time span)
SAE: Serious Adverse Event
SSNS: Steroid-sensitive nephrotic syndrome
SDNS: Steroid-dependent nephrotic syndrome
SDS: Standard deviation score
