# Supplementary material for: Randomized clinical trial to compare efficacy and safety of repeated courses of rituximab to single-course rituximab followed by maintenance mycophenolate-mofetil in children with steroid dependent nephrotic syndrome
Source: BMC Nephrol. 2020 Nov 30;21:520. doi: 10.1186/s12882-020-02153-5 (PMC7706288; doi:10.1186/s12882-020-02153-5)
Supplement: Supplementary file 1 — Additional file 1. [file 12882_2020_2153_MOESM1_ESM.docx]

**Appendix 0**

**Age specific normal reference of B lymphocyte count (n/mm^3^)**

B lymphocyte counts will be measured with flow cytometry. Our age specific normal reference of B lymphocyte count (n/mm^3^) as below

| Age | 0–3 mo | 3–12 mo | 1–2 yrs | 2–6 yrs | 6–12 yrs | 12–18 yrs |
| --- | --- | --- | --- | --- | --- | --- |
| Count | 200–1380 | 750–2250 | 510–1330 | 310–1275 | 275–640 | 175–685 |

**Appendix 1**

Baseline characteristics of the patients

| Patient’s code: |
| --- |
| Sex: |
| Age (years): |
| Weight (Kg): |
| Height (cm): |
| BMI for age Z score: |
| Height for age Z score: |
| Duration of nephrotic syndrome (years): |
| No. of relapse episodes in previous year: |
| Cumulative steroid dose in previous year (mg/kg/yr): |
| Current prednisolone dose(mg/kg/alternate day): |
| Renal Histopathology: |
| Serum albumin (g/dl): |
| Serum cholesterol (mg/dl): |
| Estimated GFR (ml/min/1.73 m^2^): |
| Urine dipstick for proteinuria: |
| Blood leucoocyte count (n/mm^3^) |
| Blood leucocyte differential count (n/mm^3^) |
| Blood Platelet count (n/mm^3^) |
| Haemoglobin (g/dl) |
| Serum Sodium (meq/l) |
| Serum Potasium (meq/l) |
| Serum Calcium (mg/dl) |
| Aspartate transaminase (IU) |
| Alanine transaminase (IU) |
| Alkaline phosphatase (IU) |
| Fasting blood glucose (mg/dl) |
| B Lymphocyte count: |
| Clinical exam: |
| Any Steroid toxicity: |

**Appendix 2**

***Adverse Event Definition***

Adverse events include suspected adverse drug reactions, other medical experiences, regardless of their relationship with the investigative drugs, such as injury, surgery, accidents, extensions of symptoms or apparently unrelated illnesses, and significant abnormalities in clinical laboratory values, or physical examination findings. Those medical conditions related to the disease under study whose changes during the study are consistent with natural disease progression, or which are attributable to a lack of clinical efficacy of the study interventions, are not considered as adverse events, however, those will be recorded in the case report forms. All other medical conditions that are present at baseline will not be considered as adverse events unless a worsening will occur.

Data on adverse events will be obtained at scheduled or unscheduled study visits, based on information spontaneously provided by the subject and/or through questioning of the participant.

***Severity of adverse events***

Adverse events will be graded according to the Common Terminology Criteria for Adverse Events, version 3.^15^ A serious adverse event is defined as an adverse event that at any dose results in inpatient care or hospital admission or significant disability or incapacity. Any serious adverse event requires expedited reporting to the sponsor safety department, regardless of its relationship to the study intervention. Hospital admissions for study procedures, or for normal disease management (relapse management) are not to be considered as serious adverse events according to this criterion. Also medically important conditions, which may not be immediately life threatening or result in death or hospitalization, but are clearly of major clinical significance, have to be considered as serious adverse events. The investigator will comply with any applicable requirements related to the reporting of serious adverse events involving his/her subjects to the Independent Ethics Committee (IEC) that approved the study and to the appropriate regulatory authority.

***Relationship and Outcome of Adverse Events***

The investigator will evaluate each adverse event that occurred after administration of the investigational drug regarding the relationship with the administration of the investigational drug:

*Definitely related:* There is a reasonable possibility that the event may have been caused by the investigational drug. A certain event has a strong temporal relationship and an alternative cause is unlikely.

*Probable:* An adverse event that has a reasonable possibility that the event is likely to have been caused by the investigational drug. The adverse event has a timely relationship and follows a known pattern of response, but a potential alternative cause may be present.

*Possible:* An adverse event that has a reasonable possibility that the event may have been caused by the investigational drug. The adverse event has a timely relationship to the investigational drug; however, the pattern of response is untypical, and an alternative cause seems more likely, or there is significant uncertainty about the cause of the event.

*Unlikely:* Only a remote connection exists between the investigational drug and the reported adverse event. Other conditions including concurrent illness, progression or expression of the disease state or reaction of the concomitant medication appear to explain the reported adverse event.

*Not related:* An adverse event that does not follow a reasonable temporal sequence related to the investigational drug and is likely to have been produced by the subject’s clinical state, other modes of therapy or other known etiology.

*Not assessable:* There is insufficient or incomplete evidence to make a clinical judgment of the causal relationship.

All subjects who have reportable adverse events, whether considered associated with the use of the study medication or not, must be monitored to determine the outcome. The clinical course of the adverse event will be followed up until resolution or normalization of changed laboratory parameters or until it has changed to a stable condition.

The outcome of an adverse event at the time of the last observation will be classified as:

*Recovered/resolved:* All signs and symptoms of an adverse event disappeared without any sequels at the time of the last interrogation.

*Recovering/resolving:* The intensity of signs and symptoms has been diminishing and/or their clinical pattern has been changing up to the time of the last interrogation in a way typical for its resolution.

*Not recovered/not resolved:* Signs and symptoms of an adverse event are mostly unchanged or worsened at the time of the last interrogation.

*Recovered/resolved with sequel:* Actual signs and symptoms of an AE disappeared but there are sequels related to the AE.

*Fatal:* Resulting in death. If there are more than one adverse event only the adverse event leading to death will be characterized as ‘fatal‘.

*Unknown*: The outcome is unknown or implausible and the information cannot be supplemented or verified.

The term “countermeasures” refers to the specific actions taken to treat or alleviate adverse events or to avoid their sequels. Following categories will be used to categorize the countermeasures to adverse events:

*None:* No action taken.

*Drug treatment:* Newly-prescribed medication or change in dose of a medication.

*Others:* Other countermeasures, e.g. an operative procedure.

**Appendix 3**

*Analysis Populations*

Full Analysis Set (FAS): includes all randomized patients who were treated with the experimental or the standard treatment at least once and they will be analyzed in the group randomized to (intention to treat principle).

Per Protocol Set: includes all patients of the full analysis set without major protocol deviations.

Safety Analysis Set: includes all randomized patients who were treated with the experimental or the standard treatment at least once, and they will be analyzed in the group as treated.
